# Foliar Symptoms Triggered by Ozone Stress in Irrigated Holm Oaks from the City of Madrid, Spain

**DOI:** 10.1371/journal.pone.0069171

**Published:** 2013-07-22

**Authors:** Carlos Calderón Guerrero, Madeleine S. Günthardt-Goerg, Pierre Vollenweider

**Affiliations:** 1 Forest Dynamics. Swiss Federal Research Institute WSL, Birmensdorf, Switzerland; 2 Department of Silvopasture, Faculty of Forest Engineering (EUIT Forestal), Universidad Politécnica de Madrid, Madrid, Spain; Portland State University, United States of America

## Abstract

**Background:**

Despite abatement programs of precursors implemented in many industrialized countries, ozone remains the principal air pollutant throughout the northern hemisphere with background concentrations increasing as a consequence of economic development in former or still emerging countries and present climate change. Some of the highest ozone concentrations are measured in regions with a Mediterranean climate but the effect on the natural vegetation is alleviated by low stomatal uptake and frequent leaf xeromorphy in response to summer drought episodes characteristic of this climate. However, there is a lack of understanding of the respective role of the foliage physiology and leaf xeromorphy on the mechanistic effects of ozone in Mediterranean species. Particularly, evidence about morphological and structural changes in evergreens in response to ozone stress is missing.

**Results:**

Our study was started after observing ozone -like injury in foliage of holm oak during the assessment of air pollution mitigation by urban trees throughout the Madrid conurbation. Our objectives were to confirm the diagnosis, investigate the extent of symptoms and analyze the ecological factors contributing to ozone injury, particularly, the site water supply. Symptoms consisted of adaxial and intercostal stippling increasing with leaf age. Underlying stippling, cells in the upper mesophyll showed HR-like reactions typical of ozone stress. The surrounding cells showed further oxidative stress markers. These morphological and micromorphological markers of ozone stress were similar to those recorded in deciduous broadleaved species. However, stippling became obvious already at an AOT40 of 21 ppm•h and was primarily found at irrigated sites. Subsequent analyses showed that irrigated trees had their stomatal conductance increased and leaf life -span reduced whereas the leaf xeromorphy remained unchanged. These findings suggest a central role of water availability *versus* leaf xeromorphy for ozone symptom expression by cell injury in holm oak.

## Introduction

Southern Europe is affected by high tropospheric ozone (O3) concentrations [1]. With 6.4 million inhabitants and 4.4 million motor vehicles, the Madrid conurbation acts as a large source of O3 precursors leading to substantial O3 pollution - especially in the Madrid outskirts [2], [3], [4], [5], [6], [7]. During the summer months on the central plateaus, the polluted air masses are re-circulated inside convective cells remaining stable for many days or even months [8], [9] making Madrid one of the regions with the highest O3 pollution in the Iberian Peninsula [3], [4], [5], [6], [7] during 2003–2008.

For several decades, visible foliar injury caused by O3 stress has been investigated in more than 75 European and 66 North American plant species and partly validated by controlled exposure experiments and microscopic analysis [6]. [11], [12], [13]. Despite a high variability, macro and micro-morphological markers of O3 stress share common structural and distribution features which can be used for identifying an O3 stress signature [14], [15], [16], [17]. These features are indicative of outbalances within the antioxidant detoxification system as a consequence of reactive oxygen species (ROS) produced in cascade after O3 uptake and synergies between O3 and photooxidative stress [18], [19], [20]. The elicited plant response and its associated structural changes in foliage can vary according to the O3 dose and levels of photooxidative stress thus leading to more than one pattern of O3 symptom expression within the same species [21]. However, O3 symptoms in broadleaved Mediterranean evergreen trees have so far seldom been documented and, to our knowledge, only one study has shown evidence of microscopic injury [22]. Holm oak (*Quercus ilex* L.) is the main tree species in many Mediterranean sclerophyll evergreen forests. Its deep rooting system, xeromorphic leaf structure and efficient stomatal control ensure tolerance to yearly summer droughts [23], [24], [25]. Compared to other sclerophylls however, it prefers rather mesic and slightly moist sites [26], [27]. In the Madrid region, holm oak is a dominant climacic species in the forest belt surrounding the city [28] and is valued as an ornamental tree in Madrid parks and streets.

The O3 sensitivity of holm oak is still controversial. In a general way and similar to other sclerophylls, this species appears to be rather O3-tolerant [1], [29], partly as a consequence of the xeromorphic traits to be found in the foliage and which are regarded as being an efficient morphological protection against O3 stress [30]. However, some of the most extreme stress reactions to O3 exposure among all sclerophyll evergreen trees so far tested were found in experiments with this species [31], [32]. Depending on the peak O3 concentration, daily irrigated holm oak seedlings thus showed photosynthesis, biomass or chlorophyll content reduction and an increase in some detoxifying enzyme activity in response to O3 exposures as low as 3.6 and 11.7 ppm•h [30], [32]. Visible leaf injury in the form of “slight stippling” [28] or “dark pigmented stipples” [31] has been observed in response to O3 exposure (AOT 40) of 59.27 ppm•h in 6 months and 79.8 ppm•h in 11 months respectively.

The present study is part of an investigation about air pollution mitigation by urban trees. During a bioindication survey, abiotic O3-like injury was identified in foliage of the holm oaks growing on an irrigated lawn strip in the center of Madrid. Given the little structural evidence available for O3 symptoms in broadleaved evergreen species, a study was undertaken in 2007 with the following objectives 1) confirm the diagnosis, 2) investigate the extent of symptoms in holm oaks growing in Madrid and 3) analyze the environmental factors contributing to O3 injury. Therefore, macro- and micromorphological markers of O3 stress were analyzed, using the aforementioned lawn strip as an intensive study site, (objective 1), 65 other urban sites with holm oaks were surveyed for similar type of leaf injury (objective 2) and data on the possible abiotic contributors, i.e. the Madrid climate, lawn strip irrigation and air pollution, were collected and analyzed (objective 3). Given the generally higher O3 sensitivity of trees growing at moist sites [33], [34] and the relative insensitivity of sclerophylls [1], [29], higher rates of stomatal conductance (first hypothesis) and reduced xeromorphic traits (second hypothesis) were hypothesized to be the principal factors determining the development of O3 injury observed at irrigated sites. Their contribution was verified by measuring gas exchanges and assessing the leaf biomass during a subsequent vegetation season (2011) at the irrigated *versus* another comparable but non-irrigated urban intensive site nearby.

## Materials and Methods

All necessary permits were obtained for the described field studies at these sites and for the symptoms survey (paragraph 2.4.) by the Madrid park service (Dirección General de Patrimonio Verde del Ayuntamiento de Madrid, signed by Mr. Santigo Soria Carrera, vice-director of green spaces and urban trees) and by the municipal authority (Departamento de Calidad del Aire del Ayuntamiento de Madrid, provided by Mr. Francisco Moya, head of the air quality department).

### Intensive Study Sites

**Figure 1:**
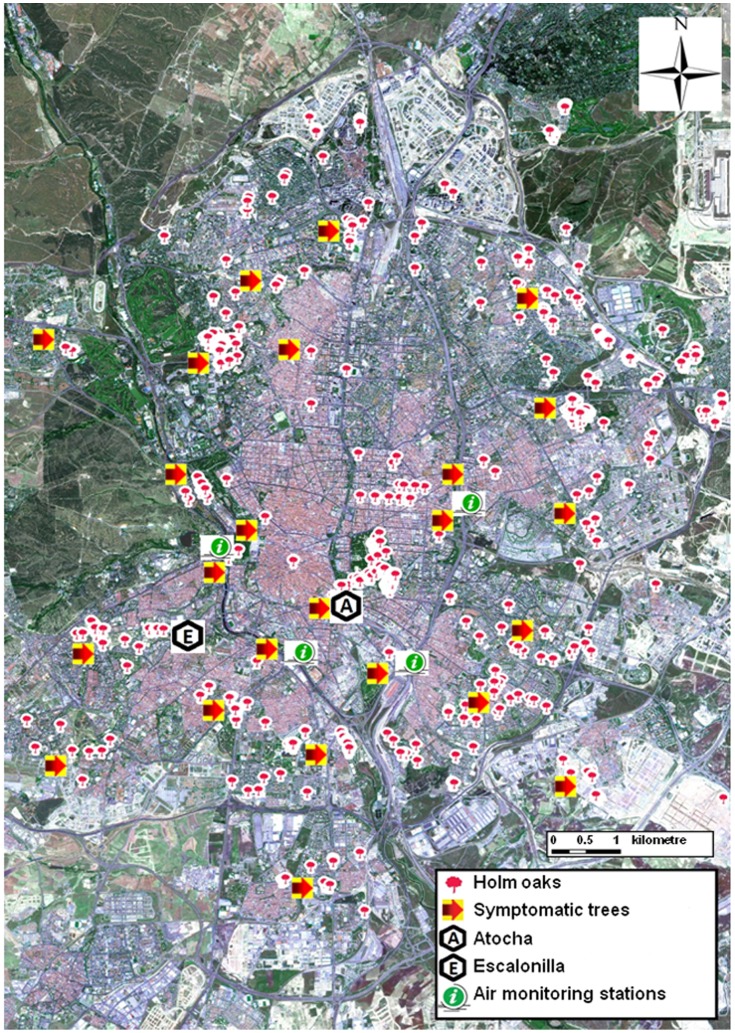
Localization of holm oak sites and air monitoring stations in Madrid. The Atocha (A) and Escalonilla (E) intensive study sites were located in the city centre. Sites with at least one symptomatic tree are indicated by red arrows.

**Figure 2:**
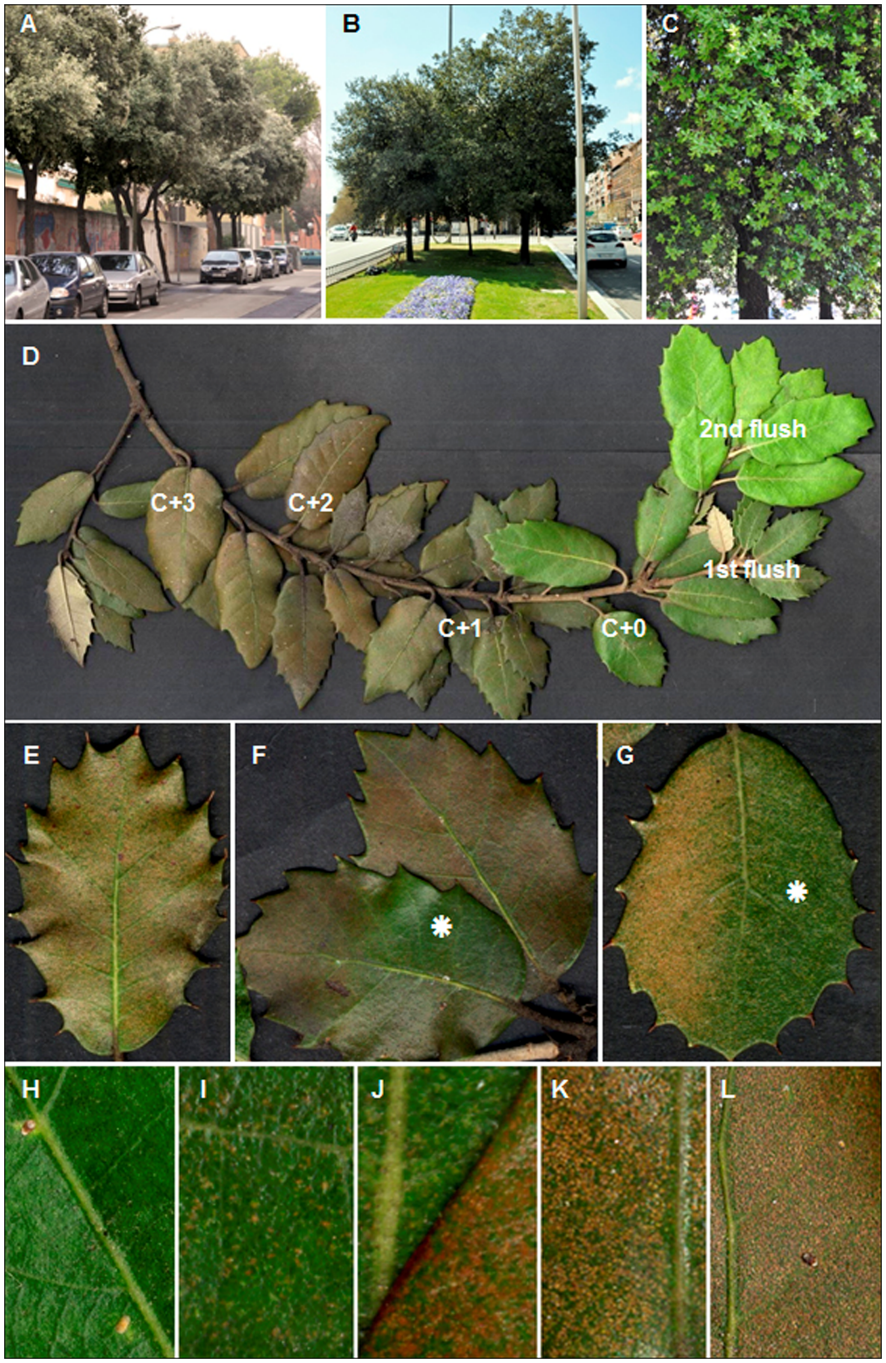
Visible injury caused by ozone stress in urban holm oaks from Madrid. **A** the non-irrigated intensive study site at Escalonilla. Trees were asymptomatic. **B**, **C** the irrigated intensive study site at Atocha. At tree level, the older and symptomatic foliage showed dark brownish tones whilst the newly flushed leaves were green (**C**). **D**–**L** visible injury in holm oak at Atocha in 2007. **D** at branch level, the symptomatic foliage showed a bronze discoloration that increased with leaf age. **E**–**L** at leaf level, symptoms were characterized by, tiny, slightly depressed, intercostal and necrotic adaxial stippling surrounded by still green leaf parts. The high stippling frequency gave an overall bronze appearance to the injured leaf (**E**, **L**). Shaded leaf parts (*) showed less injury (**F**–**G**). The stippling frequency increased with leaf age (asymptomatic: **H**: C+0; symptomatic: **I**: C+0, **J**: C+1, **K**: C+2, **L**: C+3; leaf formation: C+0∶2007, C+1∶2006, C+2∶2005, C+3∶2004).

The irrigated site was situated in the center of Madrid near the train station of Atocha (Fig. 1; Fig. 2B). It consisted of a green lawn strip irrigated by tap water sprayers and planted with *Quercus ilex* ssp. Ilex (a holm oak sub-species showing minor differences with the *Quercus ilex* spp. ballota native to the Madrid area) and with wide spaces between the trees. Escalonilla, the non-irrigated site, was situated 4 km west of Atocha along a paved street lined with similarly and regularly spaced trees planted on a 0.8 m2 grate and surrounded by a concrete pavement (Fig. 1; Fig. 2A). The holm oak subspecies at Escalonilla was the same as in Atocha.

### Climate and Air Pollution in the Madrid Conurbation

Climate conditions and air pollution of Madrid were characterized on the basis of the 1971–2007 daily records of temperature and precipitation and the 2003–2007 hourly O3 and other air pollutant concentrations from four air quality monitoring stations (Fig. 1). The closest air monitoring station was 900 m away from Atocha at a similar elevation (650 m a.s.l.). AOT40 exposure index, expressed on a daily or yearly basis, was calculated as a cumulative dose of O3 concentrations over a threshold of 40 ppb, using the April to September hourly average data measured during daylight hours and for solar radiation above 500 W/m2 [35].

SO_2_ concentrations were low over the reported period (yearly mean = 11 µg/m^3^). NO_2_ concentrations (yearly mean = 60 µg/m^3^) do not induce visible leaf injury [13], [36]. Climate, air pollution and site irrigation data was provided by the national meteorological agency (Agencia Estatal de Meteorología - AEMET), the municipal authority (Departamento de Calidad del Aire del Ayuntamiento de Madrid) and the Madrid park service (Dirección General de Patrimonio Verde del Ayuntamiento de Madrid), respectively. Irrigation data was converted to mm of precipitation per month and added to the natural precipitation to calculate the total water supply.

### Macro- and Micromorphological Observations

At Atocha, three 8 m high trees with a diameter at breast height (1.3 m, dbh) of 20±2.5 cm and with up to four leaf generations (current: C+0, 1-year: C+1, 2-year: C+2, 3-year: C+3 formed in 2007, 2006, 2005 and 2004, respectively), were selected. In June 2007, four sun-exposed branches per tree were sampled at the mid crown position, assessed for abiotic visible injury using a hand lens and dried in a herbarium after excision of leaf material for microscopy (see below). In the laboratory and to determine the percentage of stippling per leaf area, individual leaves were photographed using a macro-objective, natural light and a dark background. Digital images were analyzed by means of an image analysis system (Scion Image, Scion Corporation, Frederick, Maryland, USA) [37].

For microscopic analysis, the aforementioned sample collection harvested in June was completed in October of the same year using the same trees and mid crown branches at Atocha. Disks, 1 cm in diameter, were excised from asymptomatic and symptomatic C+0, C+1 and C+2 leaves. The leaf disks were immediately fixed either in methanol or in 2.5% glutaraldehyde buffered at pH 7.0 with 0.067 M Sorensen’s phosphate buffer. They were entirely infiltrated with the solution by evacuation before storage at 4°C until further processing. Histological, cytological and histochemical observations were performed using 2 µm semi-thin or 50 µm hand-microtomed cuttings. Semi-thin sections were obtained after dehydrating the fixed material with 2-methoxyethanol (three changes), ethanol, n-propanol, n- butanol [38], embedding in Technovit 7100 (Kulzer HistoTechnik) and cutting using a Supercut Reichert 2050 microtome. Sections were stained with different methods including toluidine blue O, *p*-phenylenediamine and acid-vanillin and subsequently mounted in inclusion medium [17], [21]. All sections were observed using a Leica microscope Leitz DM/RB, 5× to 100× objectives and diascopic light illumination. Micrographs were taken using the digital Leica DC 500 camera interfaced by the Leica DC500 TWAIN software under control of the Image Access Enterprise 5 (Imagic, Glattbrugg, Switzerland) image management system.

### Symptomatic Tree Survey

In October 2007, once all current foliage had completed its development, the extent of visible O_3_ injury in Madrid’s holm oaks was investigated by surveying 65 public park and street sites inventoried during a preceding tree survey [39]. 257 out of 2314 holm oaks with growth and age similar to trees at Atocha were selected on the basis of their dbh. Examining all leaf generations, the presence/absence of visible O_3_ injury in foliage accessible from the ground was assessed and the proportion of trees showing symptoms per site calculated. Already in the field, it quickly became apparent that symptomatic sites had been irrigated and that the mode of water supply should be recorded.

### Gas Exchange and Biomass Measurements

**Figure 3:**
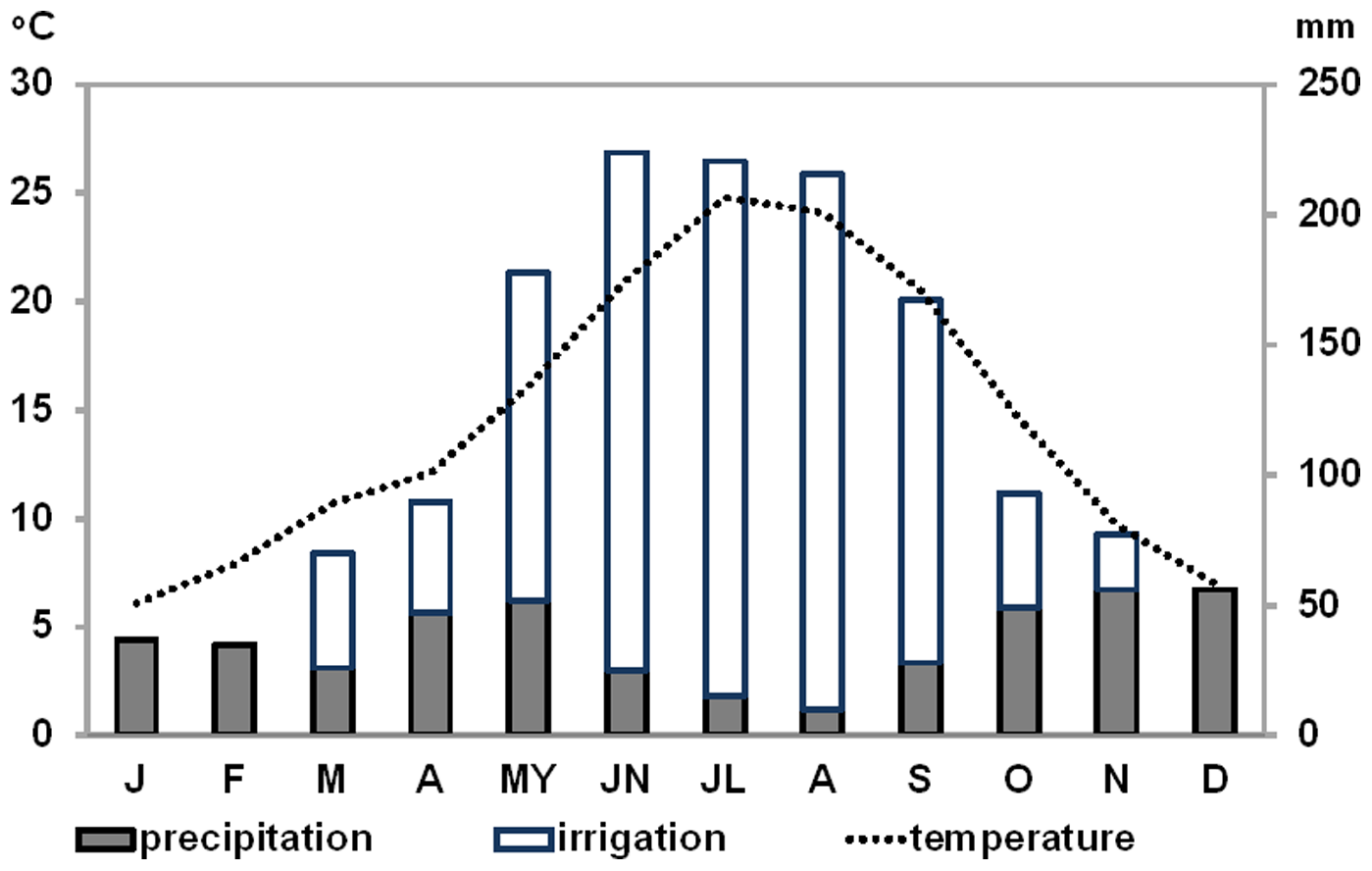
Climate diagram. Climatic conditions in Madrid and monthly irrigation totals at the Atocha intensive study site. Reference period for the climatic data: 1971–2007, average summer/winter temperature: 23.2°C/8.1°C, annual rainfall: 436 mm, annual irrigation: 1′027 mm.

**Figure 4:**
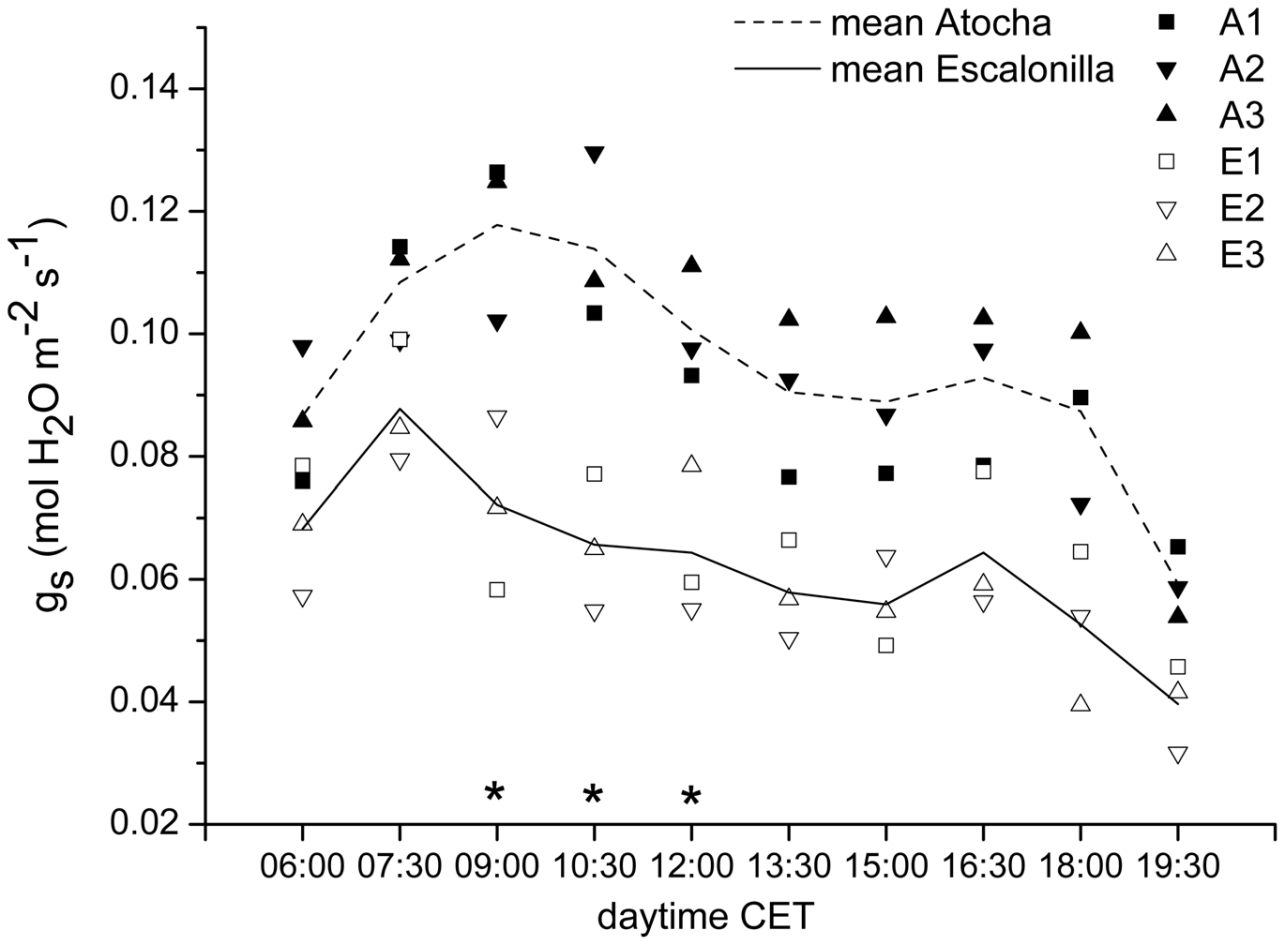
Daily time-course of stomatal conductance (gs). C+1 leaves (leaf formation: 2010) during a typical early summer day at the irrigated (A) Atocha (10^th^ of June 2011; T_min_ = 16.1°C, T_med_ = 20.1°C, T_max_ = 25.5°C) and non-irrigated (E) Escalonilla (11th of June 2011; T_min_ = 16.4°C, T_med_ = 20.5°C, T_max_ = 26°C) intensive study site (means ± SE, n = 3 trees). The factors site (p>0.0001) and daytime (P<0.003) were significant. Stars indicate a significant difference between the site means (*p*<0.05) from 9∶00 to 12∶00 am.

From February to October 2011, gas exchange and biomass measurements were carried out at the irrigated (Atocha) and non-irrigated (Escalonilla) intensive study sites using the same trees as in 2007 in Atocha and selecting trees of comparable age (40–50 years), height (8–10 m), and dbh (17.5–22.5 cm) at Escalonilla. The Atocha trees showed visible O3 injury similar to findings in 2007 with regard to the injury distribution and intensity whereas the Escalonilla holm oaks were asymptomatic. New foliage sprouted twice a year at Atocha (end of April and occasionally September, average air temperature reaching 22 and 25°C, respectively) *versus* only once at Escalonilla (end of May, average air temperature reaching 22.5°C); the leaves being completely developed by the onset of the summer drought (June, Fig. 3). Once a month, 5 leaves per leaf generation in 3 randomly selected branches (10–15 leaves per branch) from the mid part of the sun crown of each tree were measured (10 repetitions per leaf). Stomatal conductance (gs) photosynthetic active radiation (PAR) at leaf surface and leaf temperature (Tleaf) were measured in situ under ambient conditions between 09∶00 and 15∶00 (CET) using a portable infrared gas analyzer (IRGA model ADC-LCA4) equipped with a 6.25 cm2 chamber for broadleaf plants (PLC4, ADC Inc., Hoddesdon, Hertfordshire, UK). During measurements, the leaf and air temperature remained within a ±2°C range and leaf natural orientation was maintained. Daily course of gs, PAR and Tleaf was measured from dawn to dusk during two subsequent and clear days with similar weather conditions (Fig. 4), using C+1 leaves (formed in 2010).

Following measurements, the selected branches were harvested with a view to leaf area and biomass determination. Individual leaf area was ascertained using an Epson GT5000 scanner and images were analyzed using the aforementioned image analysis system. Leaves were then dried (85°C until constant weight), weighed and the leaf mass per area (LMA) determined.

### Statistical Analysis

In the case of the amount of stippling assessed at the irrigated intensive study site in 2007, an estimate for a given leaf generation was calculated by averaging measurements from three leaves per branch and four branches per tree the statistical unit being the branch (n = 4). Hence, the experiment was a split-plot design with the whole plot factor *tree* and the split-plot factor *leaf generation*. Effects of these factors on the stippling intensity were tested by means of ANOVA (with post hoc pairwise Tukey’s studentized range (HSD) test) using the SAS software package (SAS Institute, Inc, Cary NC). Given the incomplete randomization of whole plot factors in a split-plot design, the factor *tree* was tested against its interaction with the *leaf generation* factor and the *leaf generation* against the residual error.

In 2011, gs and LMA estimates per leaf generation at the irrigated *versus* non-irrigated intensive study site were calculated by averaging five leaves per branch and three branches per tree but the statistical unit in this case was the tree (n = 3). The experiment was also a split-plot design with the whole plot factor *irrigation* and the split-plot factor *leaf generation* and *month*. Effects of these factors on gs and LMA were also tested by means of ANOVA followed by post hoc tests with the factor *irrigation* and *leaf generation* tested against their interaction and the *month* against the residual error.

## Results

### Site Conditions

The climate of Madrid (Fig. 3) is Mediterranean and continental with hot summers (on average 23.2°C), cold winters (on average 8.1°C) and little annual precipitation (436 mm), especially during the summer (precipitation minimum in August). Therefore to alleviate the summer drought many places throughout the city of Madrid are irrigated either manually or automatically, as can be seen at Atocha. At this site, artificial irrigation is supplied by sprinklers at varying levels between March and November, peaking in June, July and August and reaching overall 1027 mm per year (Fig. 3).

### Ozone Pollution

**Figure 5:**
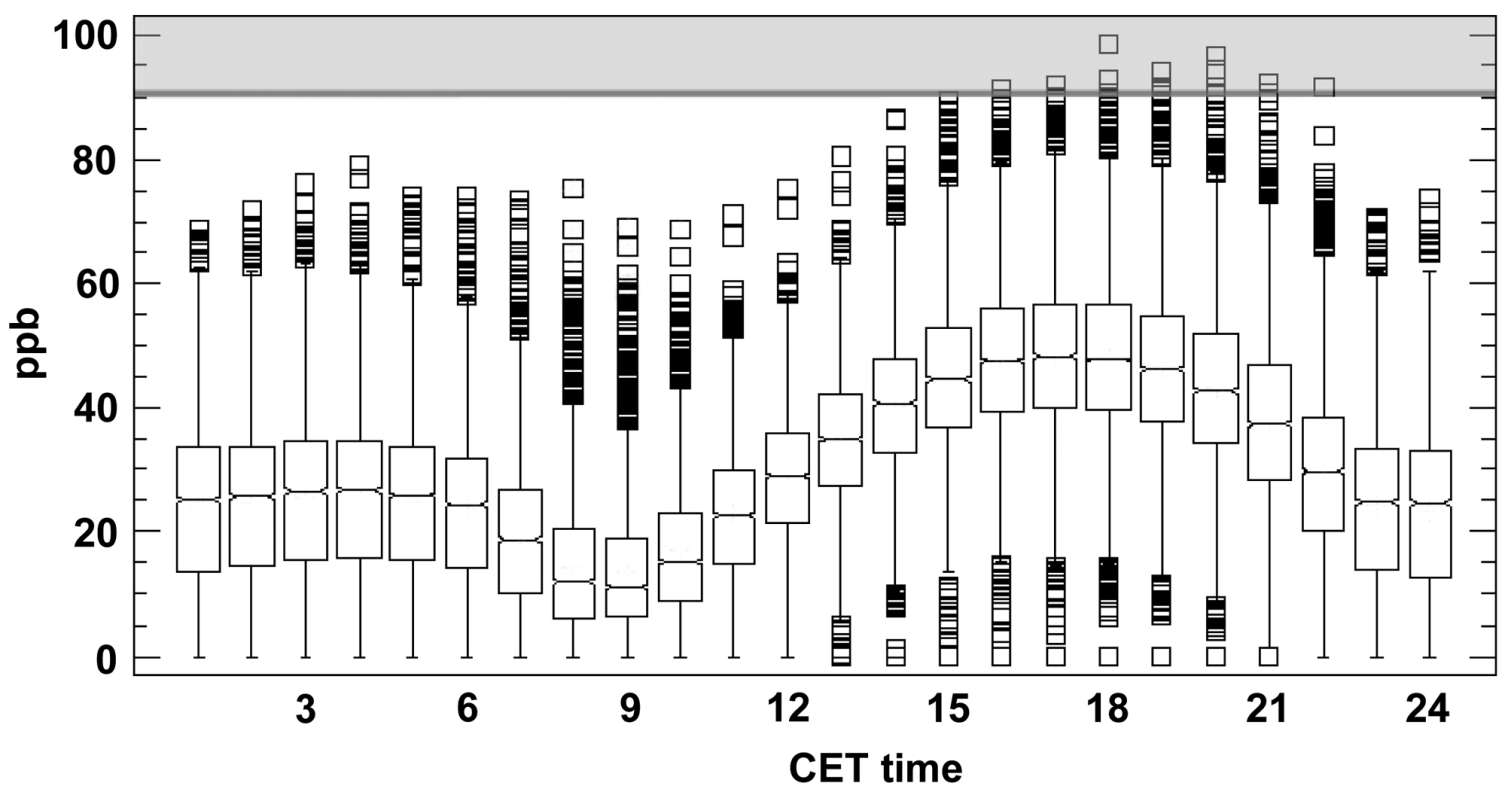
Boxplot of the average hourly (CET) O3 concentrations during the vegetation season. Data from April to September in Atocha for the years 2003–2007. The grey zone outlines the range of values exceeding the population warning threshold (box: interquartile range; whiskers: lower and upper quartiles; median horizontal line of boxes: median; white squares: maxima and minima).

The concentration and yearly course of O3 recorded at the Madrid air monitoring stations between April and September (2003–2007) was typical for an urban site. On average, and as a consequence of precursor accumulation and O3 production by road traffic and solar radiation, O3 concentration increased during the day and reached 48 ppb at 17∶00 CET. In the evening and during the night, O3 concentration dropped to a minimum of 14 ppb at 09∶00 CET (Fig. 5). The daily average, calculated on an hourly basis, reached 31 ppb with values ranging from 0 to 99 ppb. The exceedance of O3 threshold values (one-hour O3 concentration >180 µg/m3/92 ppb; [36]) amounted to 10 hours over 7 days in 2007, 13 hours over 8 days in 2006, 113 hours over 29 days in 2005, 74 hours over 22 days in 2004, and 165 hours over 36 days in 2003.

**Figure 6:**
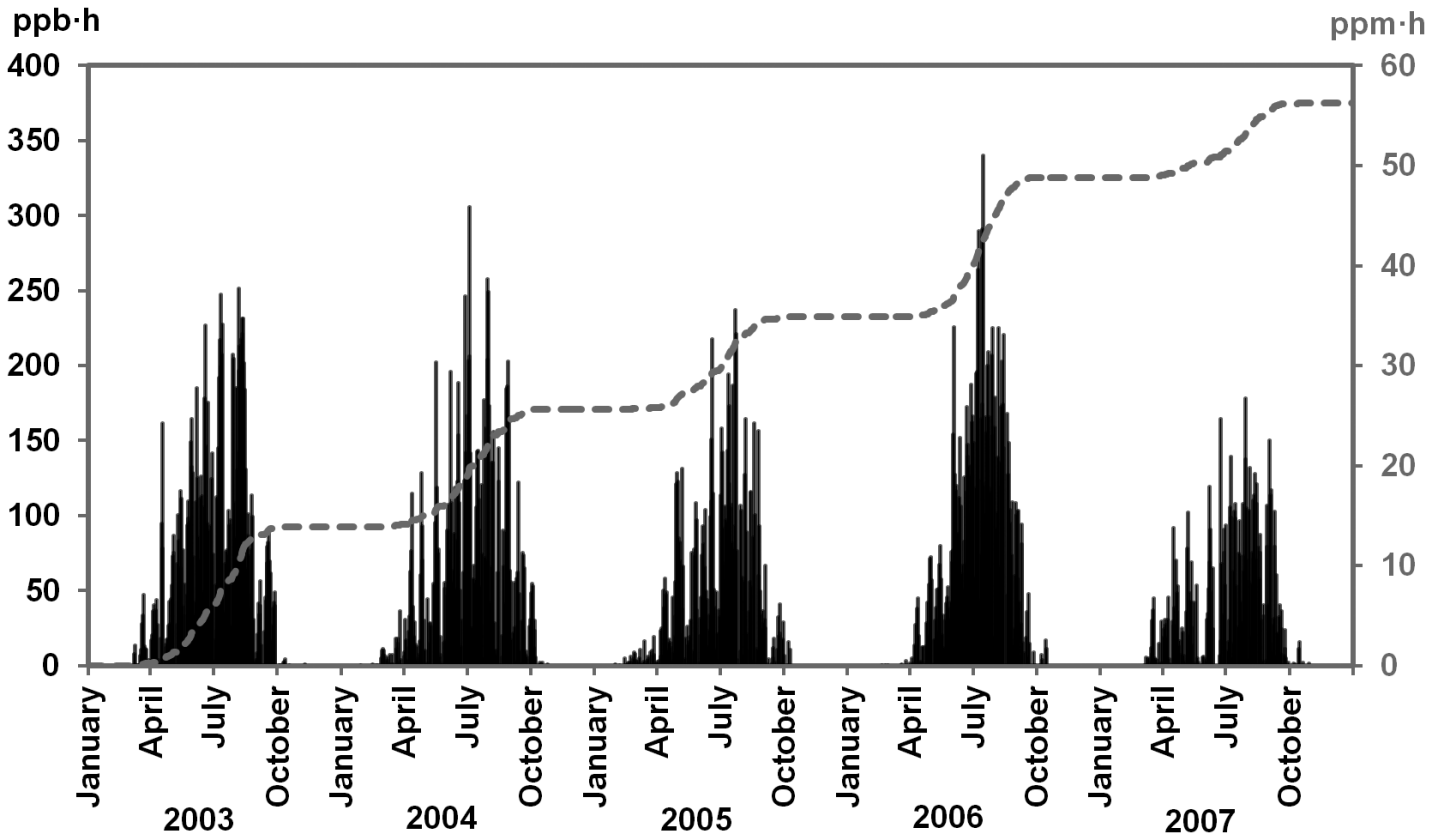
Daily (ppb•h, black spikes) and cumulated (ppm•h, grey line) AOT40 in Atocha from 2003 to 2007. Yearly AOT40 (April to September) in 2003/2004/2005/2006/2007 amounted to 14.55/11.26/9.0/13.45/7.55 ppm⋅h.

Regarding O3 exposure, yearly AOT40 (April to September) amounted to 15/11/9/13/8 ppm•h in 2003/2004/2005/2006/2007 and an average of 11 ppm•h for the whole period (Fig. 6). The highest daily AOT40 were recorded in 2003 and 2006 whereas rainy and cloudy weather, especially in 2007, reduced O3 exposure sizably. The cumulated O3 dose experienced by C+3 foliage, formed in 2004, amounted to 41 ppm•h.

### Visible Injury

**Figure 7:**
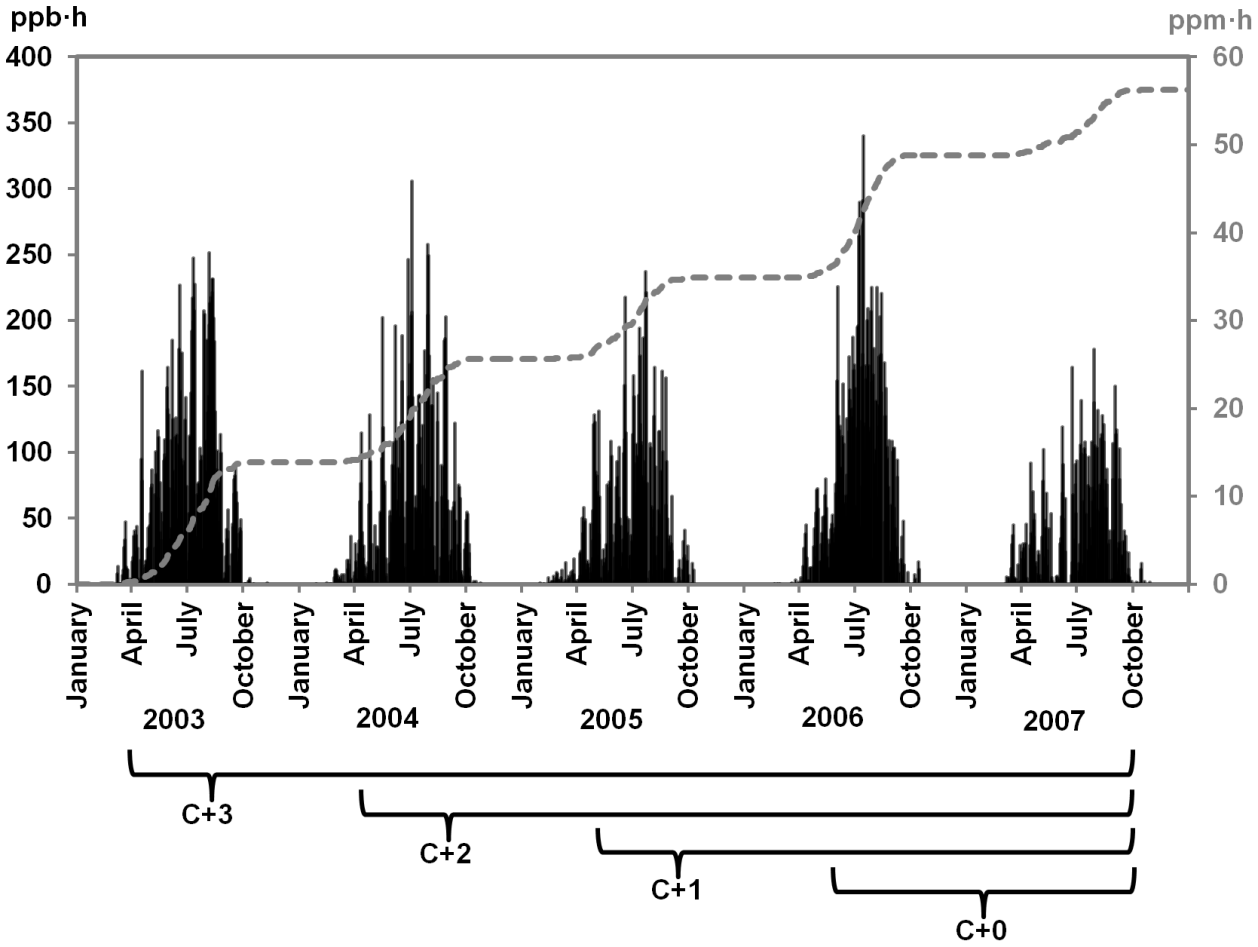
Mean percentage ± SE of leaf area showing adaxial stippling in holm oaks. Samples from Atocha in June 2007 (n = 4 branches per tree each with leaf age C+0, C+1, C+2, C+3). Different letters indicate significantly different percentages of symptomatic leaf area (*p*≤0.05).

Visible O3-like injury in foliage of holm oak appeared as depressed, tiny, necrotic and intercostal stipples amid still green leaf tissue (Fig. 2I, Fig. 2J, Fig. 2K). Their small size and high frequency let the leaf appear homogeneously discoloured unless the stipples were resolved using a hand lens (Fig. 2D, Fig. 2E *versus* Fig. 2I, Fig. 2K). Stipples developed on the upper leaf side of non-shaded foliage exposed to full sun light. Leaf parts shaded by other leaves or twigs showed reduced stippling (Fig. 2F, Fig. 2G). The recently flushed foliage (C+0; Fig. 2D, Fig. 2H) was predominantly asymptomatic whereas stipples generally developed in C+1 and C+2 leaves (Fig. 2J, Fig. 2K). Stippling intensity increased with leaf age (Fig. 2D) to such an extent as to give the older foliage an overall bronzed appearance (Fig. 2B, Fig. 2C, Fig. 2E, Fig. 2L). The stippling rates varied significantly between trees and increased with leaf age (P<0.02, Fig. 7). Holm oak subspecies showed similar type of visible injury (Fig. not shown).

Other visible symptoms occasionally observed and unrelated to the aforementioned stippling include 1) aphid exuviae and honeydew traces on C+0 leaves, 2) accumulation of soot and dust particles primarily trapped by hairs on the lower leaf side and nesting epiphytic communities in older foliage and 3) discretely distributed fungal infections (Fig. not shown).

### Microscopic Symptoms

**Figure 8:**
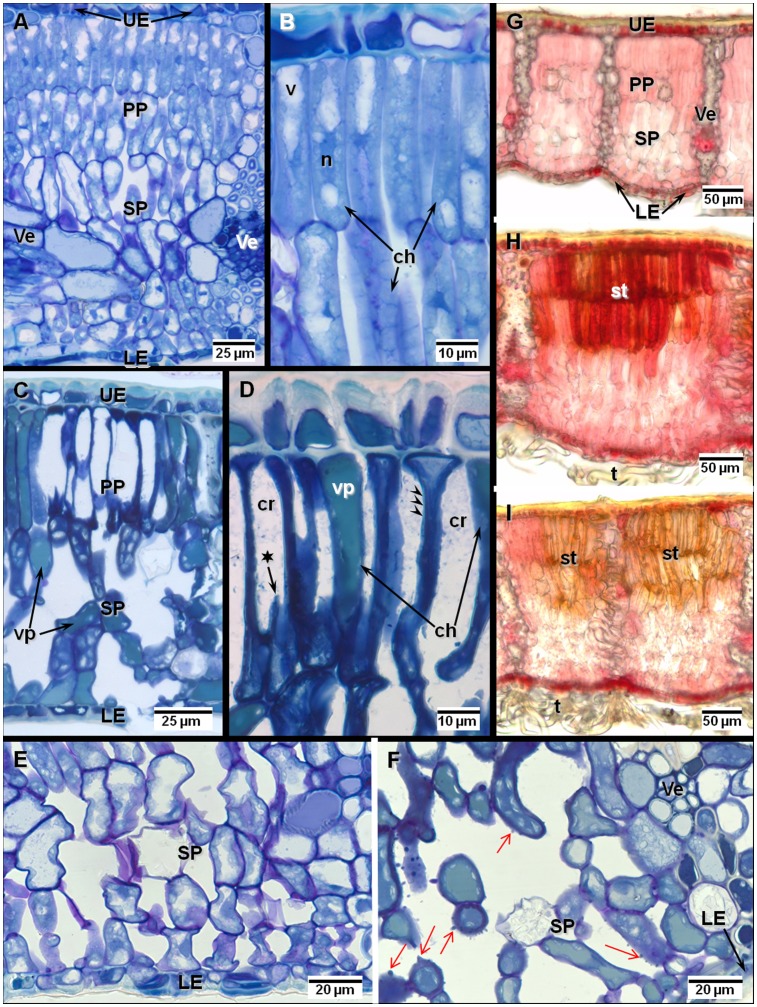
Structural and histochemical changes in the leaf blade. Leaf age/formation C+0/2007, (**G**, **H**) and C+1/2006 (**A**–**F**, **I**). Symptomatic (**C**, **D**, **F**, **H**, **I**) *versus* asymptomatic (**A**, **B**, **E**, **G**) foliar samples. Leaf parts with stipples in symptomatic *versus* asymptomatic (**C** *versus* **A**) material showed discrete groups of necrotic and collapsed palisade parenchyma (PP) cells surrounded by degenerating mesophyll tissue. At cell level (**D** *versus* **B**), necrotic cells showed cell wall thickening (arrowheads), cracking (*) and folding and a disrupted cell content. The intercellular space contained cellular remains (cr). Degenerating cells showed thickened cell walls, enlarged vacuoles (v) filled with phenolics (vp) and smaller and condensed chloroplasts (ch). Within the spongy parenchyma, cell wall protrusions (red arrows), the frequency of which increased in symptomatic versus asymptomatic material (**F** *versus* **E**), were indicative of oxidative stress in the apoplast. **G**–**I** Photo-oxidative stress in stipples (st) of symptomatic (**H**, **I**) *versus* asymptomatic (**G**) samples was shown by gradients of condensed tannin reacting with acid-vanillin (red staining) between the upper (stronger staining) and lower (weaker staining) mesophyll cell layers. In older samples (C+1, **I**) and in contrast to younger symptomatic samples (C+0, **H**), stronger oxidation of proanthocyanidins in stipples was shown by the weak reaction of condensed tannins to acid-vanillin. UE, LE upper and lower epidermis; Ve: veins; n: nucleus; t: trichomes.

The leaf blade structure of the investigated holm oak leaves showed xeromorphic traits typical of a Mediterranean evergreen tree and which include a thick leaf lamina, thick-walled and lignified epidermis, thick cuticle and lower leaf side stomata protected by a thick and dense layer of hair (Fig. 8). In leaf parts with stipples, discretely distributed groups of necrotic cells were observed in the mesophyll (Fig. 8C, D *versus* 8A, B). Necrosis developed in the upper palisade cells and often extended into the lower assimilative layers. Stipples showed characteristic hypersensitive response-like (HR-like, [41], [42]) traits including 1) distribution of dead cells in discrete intercostal groups 2) cell collapse 3) cell content disruption and 4) cell remnant condensation (Fig. 8D *versus* Fig. 8B). Similar to stipples in fumigated foliage of *Fraxinus ornus* [21], folds and cracks in cell walls together with cell fragments leaking into the intercellular space were observed. Stipples were surrounded by degenerating cells as shown by cell wall thickening, chloroplast condensation and vacuolar accumulation of phenolics (Fig. 8C). Interestingly, the latter two markers were also observed within dead cells belonging to stipples (Fig. 8D). In contrast to the tissue level, cell-level gradients of injury caused by varying light exposure were missing. Droplets of cell wall material protruding into the inter-cellular space were often observed in the lower leaf blade - mostly within the spongy parenchyma layers (Fig. 8F *versus* 8E). Elevated levels of oxidative stress were indicated by the accumulation of oligo-proanthocyanidins (OPC) inside and surrounding recently formed stipples (Fig. 8H *versus* Fig. 8G). In C+1 *versus* C+0 leaves, an increase in oxidation of the cellular material was shown by the lower OPC signal and brownish unspecific staining of stipples (Fig. 8I *versus* Fig. 8H).

Structural changes by fungi and bacteria or insects were detected but they were spatially distinct and causally unrelated to stipples. They included 1) cell wall thickening in cells of hairs covering the lower leaf side and trapping dust particles or 2) cell collapse, cell wall thickening and cell content degeneration in vein phloem and nearby lower leaf blade tissues as a consequence of aphid feeding (Fig. not shown).

### O3 Symptoms Survey

Out of the 65 sites surveyed in the Madrid conurbation in 2007, 24 (37%) including Atocha, were symptomatic with foliage of holm oaks showing varying levels of O3 injury (Fig. 1). With the exception of one site next to a stream, all symptomatic sites had supplementary water supplied by an automated irrigation system. On average, the proportion of symptomatic trees per site amounted to 25% ±5.3 (SE; range: 11–100%). Out of the 41 asymptomatic sites, only 12 (30%) were irrigated with water supplied by drip (19%) or manual (11%) once a month, presumably with lower amounts than at symptomatic sites. Variation in the plot size, water supply, site conditions or holm oak sub-species prevented further quantification of the stippling frequency.

### Stomatal Conductance

On average, during a typical summer day in 2011, and from dawn to dusk, the C+1 holm oak foliage showed higher gs at Atocha than Escalonilla (P<0.001) with values 55.5% larger at the irrigated *versus* non-irrigated intensive study site (Fig. 4). At both sites, gs was highest in the morning topping at 9∶00/7∶30 CET in Atocha/Escalonilla. Gs experienced a slight midday depression increasing moderately again from 16∶00 to17∶00 CET, prior to a further drop in the evening. Hence, at Atocha, besides increasing gs, irrigation delayed the midday depression by a few hours, as shown by significant differences (P = 0.05) between the sites from 9∶00 to 12∶00 am (Fig. 4).

**Figure 9:**
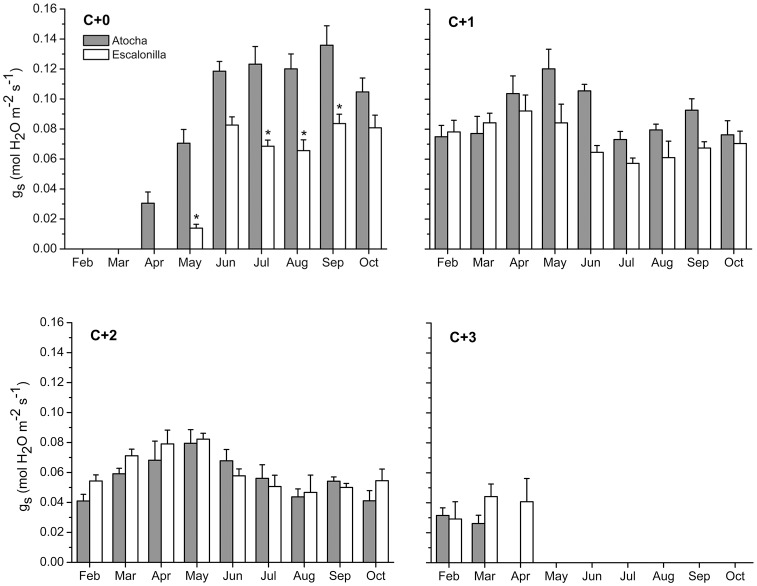
Seasonal variation of stomatal conductance (gs). C+0, C+1, C+2 and C+3 foliage (leaf formation: 2011, 2010, 2009, 2008, respectively) at the irrigated (Atocha, grey bars) *versus* non- irrigated (Escalonilla, white bars) intensive study site in 2011 (means ± SE, n = 3 trees). The monthly irrigation supply at Atocha is shown in Figure 2, the significance of influencing factors in Table 2.

The stomatal conductance varied as a function of the irrigation, leaf age and month (Table 1; Fig. 9). Particularly current year leaves C+0 followed the irrigation curve (Fig. 9 compared to Fig. 3, Pearson correlation coefficient for C+0 and irrigation = 0.88), whereas C+1 was correlated to C+2 (0.86). The site irrigation caused a significant increase in gs from May to October. During the peak irrigation period (May to September), gs values in C+0/C+1 leaves were 54%/31% higher on average at Atocha *versus* Escalonilla. Leaf age caused a significant decrease in stomatal conductance and younger leaves were more responsive to higher water availability (significant irrigation*leaf age interaction from May to October, Table 1). The C+1 and C+2 leaves showed a decrease in gs after the new C+0 foliage had sprouted. During the vegetation season (February to October), stomatal conductance varied between months, especially regarding younger leaves (significant leaf age*month interaction from May to October).

**Table 1:** Significance (P-values) of two-way analysis of variance.

Factor	d.f.	g_s_ February to April	g_s_ May to October
Irrigation	1	ns	<0.001
Leaf age	3	0.001	<0.001
Month	2 or 5	0.011	ns
Irrigation * leaf age	2	ns	0.003
Irrigation * month	2	ns	ns
Leaf age * month	4 or 10	ns	0.006

Effects of the factors: irrigation (Atocha *versus* Escalonilla), leaf age/formation (C+0/2011, C+1/2010, C+2/2009, C+3/2008) and month on stomatal conductance (gs) and their interactions during spring with little irrigation (February to April) and summer with irrigation (May to October), ns not significant p≥0.05.

### Leaf Biomass Partition and LMA

**Figure 10:**
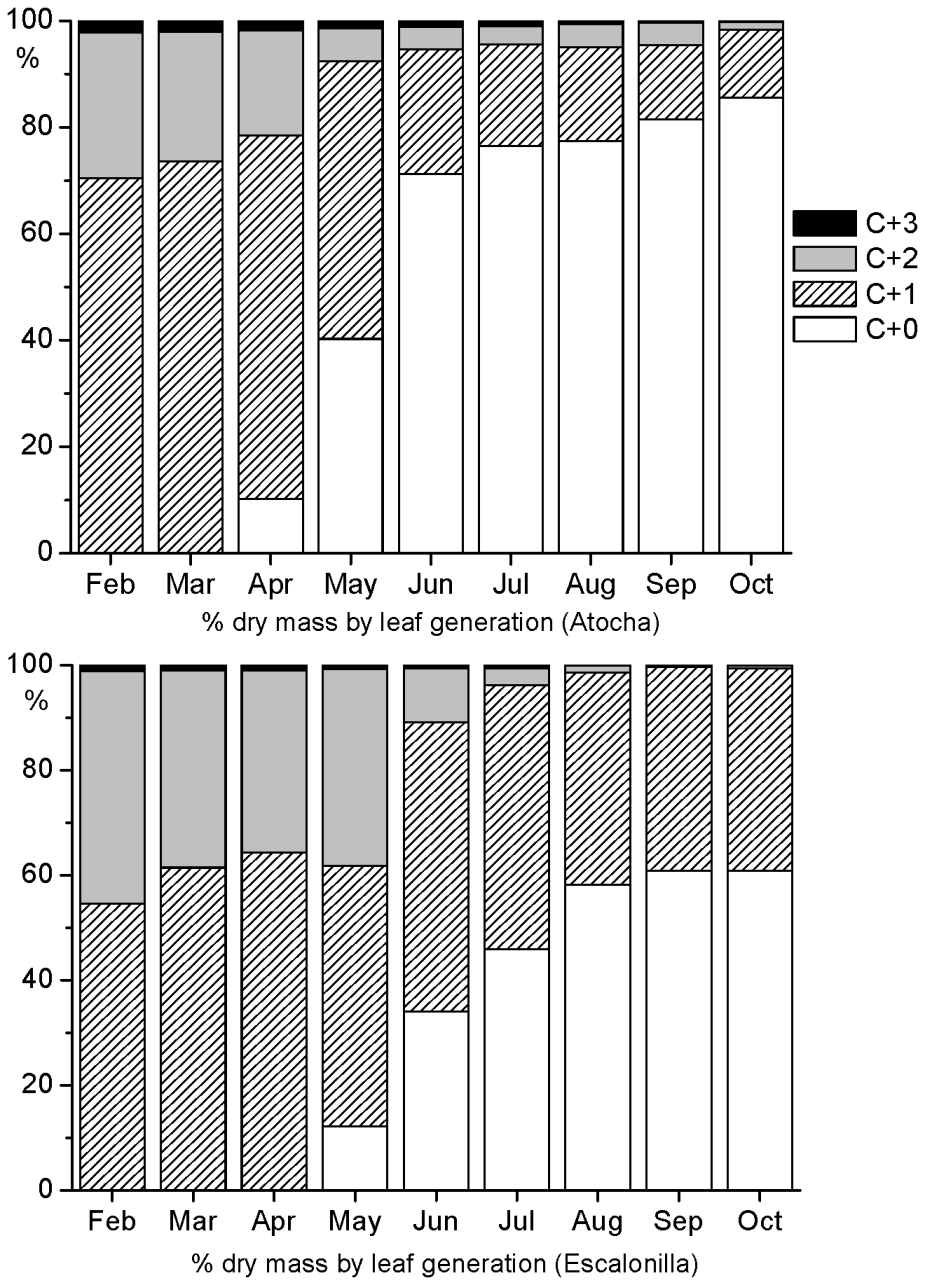
Monthly changes in the foliage biomass fraction (expressed in %) of each leaf generation. Leaf age/formation C+0/2011, C+1/2010, C+2/2009, C+3/2008 within holm oaks from the irrigated (Atocha) and non-irrigated (Escalonilla) intensive study site in 2011 (mean values of 3 trees).

At both intensive study sites, the youngest leaf generation formed the highest biomass fraction in the analyzed branches (Fig. 10). However, there were differences between sites and, prior to and after the development of new C+0 leaves, older foliage made up a larger proportion of the total foliage biomass at Escalonilla than at Atocha. Hence from June to October 2011, during the highest irrigation and most O3-polluted period, the C+0 and older foliage biomass fraction amounted to 56% and 44% at Escalonilla *versus* 80% and 20% at Atocha. Finally at each site in October, the C+0 foliage’s contribution to the total foliage biomass amounted to 61% and 86%, respectively.

As indicated by LMA and irrespective of the leaf generation, holm oak foliage showed a similar leaf xeromorphy at both sites (Table 2). During the 2011 vegetation season, monthly LMA estimates for C+1 and C+2 leaves at Atocha *versus* Escalonilla were not significantly different except during new foliage development (April and May). Regarding the C+0 leaves, it took three months at Atocha *versus* four at Escalonilla (Fig. 10) until adult and comparable leaf LMA values could be achieved (75±10.5 to 162±5.3 mg/cm2 from April to June *versus* 74±1.3 to 164±3.6 mg/cm2 from May to July).

**Table 2:** Mean leaf mass per area.

Leaf age/formation	C+0/2011	C+1/2010	C+2/2009	C+3/2008
Atocha	16.5±0.1	16.9±0.3	17.3±0.1	17.2±0.3
Escalonilla	16.5±0.1	16.7±0.3	17.1±0.2	17.1±0.3

LMA (mg/cm2) ± SE per leaf generation at the irrigated (Atocha) and non-irrigated (Escalonilla) site in October (C+0, C+1, C+2) and March (C+3) 2011. Differences between sites and leaf generations were not significant (p>0.05; N = 3 trees).

## Discussion

### Stipples as Structural Injury Due to Ozone Stress

Within the analyzed holm oak leaves, the stipple morphology and the changes observed at cell level were typical of those associated with acute O3 stress as described for deciduous broadleaved species [13], [43]. Apparently, the thick leaf blade, a xeromorphic trait in *Q. ilex* [44], [45], did not modify the development of stipples although the distance between the O3-absorbing stomata and necrotic upper palisade parenchyma was larger than in deciduous foliage. In comparison to *Pistacia lentiscus* [22], not only degenerative changes but also necrotic stipples indicative of HR-like and resulting from defensive programmed cell death (PCD, [13], [46]) were found. Cracks in cell walls and cell content leakage similar to HR-like injuries reported for fumigated Manna ash seedlings [21] indicated a large production of and severe injury by ROS [47]. Differing from the latter species, necrotic cells in the present study showed phenolic accumulation and cell content disruption suggesting that PCD was preceded by a degenerative phase lasting more than a year according to morphological observations about stippling emergence. Indeed, PCD is ROS- concentration dependent [48] and an oxidative stress threshold thus needs to be exceeded prior to activating a PCD-program. Other oxidative and O3 stress markers in the studied holm oaks included 1) the wart-like droplets in lower mesophyll [49], [14], [16], 2) the positive reaction with acid-vanillin in and around young stipples [50] and 3) the impediment of the acid-vanillin reaction within older leaf material due to the oxidation of necrotic cell remnants [17]. The interaction between O3 and photo-oxidative stress [21] was indicated by the gradient of injury and OPC between lower and upper mesophyll in leaf parts with stipples.

The visible stippling morphology and distribution, together with the observed shading effects, were typical for O3 stress [10]. The homogeneous and intercostal distribution of stipples in foliage of the sun-exposed crown, their frequency increasing with leaf age and their occurrence within large tree crown portions of several trees per site at the many sites further confirmed the diagnosis [51]. Regarding the role of other stress factors, as potential causes for the observed leaf injury, besides ozone, a contribution can be excluded based on: 1) other phytotoxic components of photochemical smog, such as peroxyacetyl nitrate (PAN), do not cause HR-like reaction leading to stippling symptoms [13], [52], 2) the concentration of other gaseous air pollutants, such as the aforementioned SO_2_ and NO_2_, were too low or not phytotoxic, 3) the detected biotic injury was spatially and causally not related to the analyzed stippling, 4) eventual nutrient deficiencies or imbalances cause specific patterns of visible injury different from those caused by ozone stress [43] and 5) eventual soil contamination with metals lead to microscopic changes primarily along the water pathway through the leaf and these microscopic symptoms are clearly different from those induced by ozone stress [13], [51].

Ozone-triggered stippling has already been observed in fumigated holm oak seedlings [31], [32] and similar visible leaf injury has been documented in other deciduous and partly evergreen Spanish oak species (*Q. faginea, Q. pyrenaica*) exposed to O3 under controlled conditions [53]. To our knowledge however, the findings presented here are the first to show the structural changes associated with O3-triggered stippling in leaves of holm oak.

### Ozone Stress in the Holm Oaks of Madrid

In the center of Madrid, the 2003–2007 AOT40 average (11 ppm•h) was above both the former (10 ppm•h) and present (5 ppm•h) concentration-based critical level for European forest trees [54], [55]. Compared to other South-Western European sites, it was slightly inferior to the 2000–2002 AOT40 range (13–19 ppm•h), whereas the warmer 2003 and 2006 years fitted into the lower part of the range [56]. Gradients of O3 concentration, increasing between the Madrid center and suburbs and varying according to micro-climatic conditions [2], may relate to the higher frequency of symptomatic sites at the city’s periphery. Between 2003 and 2007, Madrid experienced exceedances over the 180 µg/m3 alert threshold more often than on average in the Iberian Peninsula but less frequently than in South-Eastern France and Italy (for example [3], [4], [5], [6], [7], 2003–2008). The 21 ppm•h O3 exposure required for the appearance of visible injury in holm oak at Atocha (sum of 2007 and 2006 AOT40) was lower than that indicated for the fumigated holm oak seedlings mentioned previously [31], [32]. However, it was still largely higher than that which causes symptoms in most young deciduous broadleaved trees so far tested [57], [58], [59] or promoting functional alterations in holm oak foliage fumigated experimentally (3 ppm•h, [32]). The O3 exposure needed for the development of leaf injury may also change according to the years and experimental settings as shown for *Pistacia lentiscus*, another evergreen xerophyte, with injury threshold varying from 9 to 74 ppm•h [22]. Hence, the O3 exposure recorded at Atocha was considerable with respect to the Iberian average but remained, with regard to structural injury in holm oak, within the lower range of values expected to cause symptoms in a sclerophyll evergreen tree relatively insensitive to O3 stress [1], [60].

### Increased Ozone Uptake in Holm Oak Foliage as a Trade-off for Site Irrigation

By raising gs in Atocha *versus* Escalonilla, irrigation was confirmed to increase O3 uptake during the whole day and alleviate the midday gas exchange reduction during peak O3 hours. Other studies have also documented the responsiveness of holm oak to higher soil moisture availability [61], [62] with enhanced O3 stomatal uptake at O3 polluted sites as a trade-off for an elevated water supply [63], similar to findings on other species [33]. In Madrid, findings from the leaf injury survey suggest that O3 symptoms were even conditioned to site irrigation and higher water availability. At Atocha, the highest levels of O3 exposure, site irrigation and leaf gs were recorded during the summer and these factors could synergistically contribute to an increased O3 uptake in irrigated *versus* non-irrigated holm oak. Interestingly, only the younger C+0 and C+1 leaf generations were responsive to elevated water availability. Besides shading by new foliage and lower gs with increasing leaf age [64], leaf injury might further reduce gs in older holm oak foliage as suggested by the concomitant development of stippling and reduction of gs in C+1 leaves recorded during the summer. Overall, the gs values measured in Madrid were in the range of those published for Mediterranean forests subjected to summer drought [65], [66], the highest gs rates for C+0 leaves were in line with findings by [62]. The C+0 and C+1 leaf generations with the highest gs were also those least symptomatic. This paradox probably related to the aforementioned exceedance of an oxidative stress threshold needed for triggering a PCD-program and causing visible stippling, as a consequence.

### Leaf Life Span of Irrigated Foliage

Compared to Escalonilla, the leaf turn-over in Atocha’s holm oak foliage was accelerated. Generally, foliage showing higher stomatal conductance, as in Atocha, is also shed earlier [67]. Furthermore, water-restricted versus water unrestricted evergreen trees tend to keep their older foliage a longer time and use it more intensively [68]. Competition between older and younger leaves might also contribute to leaf turn-over, as suggested by leaf-drop primarily after new leaf flushing instead of throughout the spring and after the summer drought period, as usual. Besides leaf physiology and competition factors, O3 accelerates leaf senescence [69], [70] which can lead to a reduction in the amount of leaf generations in evergreen trees [71]. Here, this effect is suggested by the concomitant decrease of gs and development of stippling. Hence and synergistically with other causes, O3 might contribute to reduced leaf life span in the irrigated and symptomatic holm oaks.

### Leaf Xeromorphy and Irrigation

Whatever the leaf generation, the leaf xeromorphy was not affected by irrigation, as indicated by similar LMA at both study sites. With reference to [72], these findings were unexpected. The values found in Madrid were similar to those indicated for holm oak in other urban conditions [73] on rather mesic Italian sites [74] or under similar precipitation and temperature regimes in Catalonia, Spain [75]. As found by [45], the LMA of mature leaves did not change significantly through time. Consequently, the development of O_3_ injury proceeded independent of the leaf xeromorphy and primarily related to enhanced gs and higher O_3_ uptake.

### Conclusions

In synthesis, the initial O3 symptom diagnosis was confirmed on the basis of the macro- and micro-morphological changes found in irrigated holm oak foliage (objective 1). Ozone injury similar to that detected at the intensive study site of Atocha was found throughout Madrid but predominantly at sites with automated irrigation (objective 2). O3 exposure up to a harmful level for the natural vegetation was recorded in air monitoring stations close to our intensive study sites but at levels apparently too low to cause visible injury in an evergreen tree rather insensitive to O3 stress (objective 3). On the basis of subsequent gas exchange and biomass/LMA measurements, higher rates of stomatal O3 uptake in irrigated and symptomatic trees were corroborated (first hypothesis) whereas no difference in the leaf xeromorphy between the irrigated and non- irrigated site was found and therefore the second hypothesis was rejected. Given the concomitant maximum irrigation and peak O3 pollution, the O3 tolerance of irrigated holm oaks appeared to be lowered to levels similar to those recorded for other broadleaved trees. This particular case of leaf injury by O3 stress because of the irrigation gives insight into mechanisms driving O3 symptom expression in sclerophyll evergreen trees. In holm oak at least, they suggest that stomatal closure, particularly during peak O3 pollution, can be more effective than leaf xeromorphy to reduce stomatal O3 uptake and outlines the driving contribution of soil moisture availability for O3 symptom expression in dry climates.
