# Correction: Foliar Symptoms Triggered by Ozone Stress in Irrigated Holm Oaks from the City of Madrid, Spain

**DOI:** 10.1371/annotation/d10ff5cd-1c4b-4d18-9735-4929108d4398

**Published:** 2014-01-02

**Authors:** Carlos Calderón Calderòn Guerrero, Madeleine S. Günthardt-Goerg, Pierre Vollenweider

Figure 7 is incorrect. Please see the correct Figure 7 here: 

**Figure:**
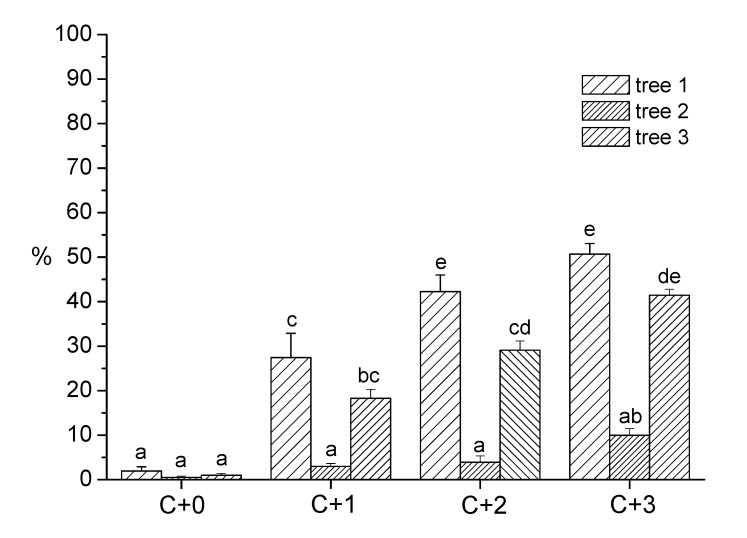


In addition, the name of the first author was incorrectly expressed in the Citation. The correct Citation is: Calderòn Guerrero C, Günthardt-Goerg MS, Vollenweider P (2013) Foliar Symptoms Triggered by Ozone Stress in Irrigated Holm Oaks from the City of Madrid, Spain. PLoS ONE 8(7): e69171. doi:10.1371/journal.pone.0069171. 

